# Visualizing nociplastic pain: functional hyperexcitability in neuropathic and idiopathic facial pain syndromes

**DOI:** 10.1186/s10194-025-02133-w

**Published:** 2025-10-13

**Authors:** Arne May, Vanessa Ciancia, Hauke Basedau

**Affiliations:** 1 Department of Systems Neuroscience, University Medical Center Hamburg- Eppendorf, Martinistr. 52, D-20246 Hamburg, Germany; 2 Department of Oral and Maxillofacial Surgery, University Medical Center Hamburg-Eppendorf, Hamburg, Germany; 3 Center for Anesthesiology and Intensive Care Medicine, Department of Anesthesiology, University Medical Center Hamburg- Eppendorf, Hamburg, Germany

**Keywords:** Neuropathic facial pain, Trigeminal nerve, Brainstem imaging, FMRI, Pathophysiology

## Abstract

**Background and objectives:**

Neuropathic facial pain (NFP) is linked to lesions of trigeminal nerves, distinguishing it from persistent idiopathic facial pain (PIFP) which is clinically similar but without neurological abnormalities and without clinically objectifiable cause. Our recent brainstem imaging studies have demonstrated that abnormal central pain processing plays a role in the pathophysiology of PIFP.

**Methods:**

Using the identical brainstem imaging protocol this current study aims to investigate the trigeminal pain processing mechanisms in patients with peripheral NFP using functional magnetic resonance imaging (fMRI).

Twenty patients with NFP and 20 healthy control subjects (HC) participated in a well-established protocol of trigeminal nociceptive stimulation using gaseous ammonia. Functional images were acquired with a 3T MRI scanner, utilizing an optimized protocol designed for high-resolution echoplanar imaging of the brainstem. In a further step, using the same protocol, the imaging data from our database of patients with PIFP characterized by specific clinical features of persistent facial pain without detectable neurological damage, were incorporated into the analyses.

**Results:**

Patients with NFP exhibited stronger activation in the spinal trigeminal nucleus (sTN) and other pain transmitting areas in response to painful stimulation when compared to HC. This finding is similar to a previous study in PIFP patients using the same imaging parameters. When contrasting patients with PIFP from this earlier study with NFP patients, a network of overactive brain areas further distinguished PIFP patients.

**Discussion:**

Our findings indicate that altered central pain processing contributes to the pathophysiology of PIFP, possibly as a sign for nociplastic changes. Integrating these results into facial pain models could enhance the overall understanding of mechanisms underlying NFP and PIFP.

**Supplementary Information:**

The online version contains supplementary material available at 10.1186/s10194-025-02133-w.

## Introduction

Neuropathic facial pain is defined by chronic pain associated with lesions of trigeminal nerves [2, 3], often resulting from trauma, surgical procedures, or other nerve-injuring events, such as herpes zoster infection [4]. These identifiable causes set neuropathic facial pain apart from persistent idiopathic facial pain (PIFP), a condition where the chronic facial pain has no apparent neurological or anatomical origin [5]. The distinction between these two types of facial pain, which are clinically otherwise identical, is critical, as it may underscore differing underlying pathophysiological mechanisms.

Neuropathic pain may be divided into peripheral, central or mixed (peripheral and central) types. As defined by the International Association for the Study of Pain (IASP), neuropathic pain results from a lesion or disease of the somatosensory nervous system. Three levels of certainty have been established: possible, probably and definite [6, 7]. The grades “probable” and “definite” require confirmatory evidence from a neurologic examination, such as tests indicating negative or positive sensory signs in the innervation territory of the affected nerve and diagnostic tests confirming a lesion.

In neuropathic facial pain, peripheral nerve damage may subsequently lead to central pathways being activated or altered, interpreted as sensitization. This may involve the aberrant processing of pain signals, increased excitability of neurons, or altered connectivity within ascending trigeminal pain pathways [4]. Conversely, PIFP is more obscure, as it presents with persistent pain but no distinct nerve damage. This lack of identifiable lesions despite the constant pain suggests that PIFP mechanisms may primarily involve alterations in central nervous system pathways [1, 8, 9].

In this context, our study aims to elucidate the distinct features of trigeminal pain processing at the brainstem level in patients with probable NFP. By leveraging functional magnetic resonance imaging (fMRI), we aimed to map the specific alterations in pain processing pathways characterizing NFP, as opposed to PIFP.

## Materials and methods

The study was conducted following approval from the local ethics committee of the Chamber of Physicians in Hamburg, Germany (PV4762), and followed the principles outlined in the Declaration of Helsinki. Written informed consent was obtained from all participants, who retained the right to withdraw from the study at any time. This study was preregistered on October 21, 2020 via the Open Science Framework (https://osf.io/62dq4/). The study adheres to the STROBE Statement [10].

### Study participants

Participants were recruited from the Facial Pain Center of the University Medical Center Hamburg-Eppendorf, Germany. Patients are usually referred by neurologists, dentists, and general practitioners after thorough exclusion of possible secondary pain syndromes. Those diagnosed with neuropathic facial pain were selected based on established diagnostic criteria evidencing nerve-related lesions [6, 7]. As definite neuropathic pain requires the ancillary test confirming a trigeminal nerve lesion, which is not routinely conducted in our center, we targeted patients diagnosed with probable neuropathic pain, characterized by persistent facial pain of burning quality confined to the second or third trigeminal branch on one side, and presenting with negative (hypoesthesia) or positive (allodynia, paresthesia, etc.) sensory signs in the innervation territory of the pain area. Criteria for inclusion encompassed the confirmed neuropathic diagnosis and the willingness to participate in the research. Exclusion criteria ruled out individuals with concurrent primary or secondary headache disorders, contraindications to MRI procedures, or other psychiatric or neurological conditions. All patients had additionally normal sum scores for depression, addiction and anxiety in the Patient Health Questionnaire (PHQ). Age- and sex-matched healthy controls (HC) met the same exclusion criteria as the patients and were primarily recruited via advertisement on a web portal of the University of Hamburg.

In a further step, the imaging data from our database of patients with PIFP [1], characterized by specific clinical features of persistent facial pain without detectable neurological damage, were incorporated into the analyses. The exclusion criteria for PIFP patients were also identical to the NFP patients and ruled out individuals with concurrent primary or secondary headache disorders, contraindications to MRI procedures, or other psychiatric or neurological conditions. All patients had additionally normal sum scores for depression, addiction and anxiety in the Patient Health Questionnaire (PHQ).

We have based our sample size on multiple experiments that we have done using this validated brain-stem optimized functional imaging study design [11–15] and have mentioned this in our preregistration in 2020.

### Experimental design

All participants underwent an event-related fMRI session wherein they received a series of 15 painful stimulations to the trigeminal nerve using intranasal gaseous ammonia, interspersed with control stimuli such as air puffs, rose odor, and visual stimulations [11]. The stimuli were presented in a pseudorandomized order to prevent adaptation or anticipation effects that could bias results. The detailed stimulation paradigm has been described extensively by our group [11, 15–18]. Participants rated the perceived intensity of each stimulus in the scanner using a visual analogue scale, allowing for subjective comparisons of pain intensity.

### MRI acquisition and analysis

#### MRI acquisition

For this study, images were collected using the Siemens PRISMA 3 Tesla MR system (Siemens, Erlangen, Germany). The echoplanar imaging utilized a specialized protocol to achieve high-resolution images of the brainstem [1]. This protocol included small voxel sizes of 1.25 × 1.25 × 2.5 mm³, with a repetition time (TR) set to 2.61 s and an echo time (TE) of 27 milliseconds. We captured 38 axial slices, working with a field of view (FoV) of 216 × 108 mm², aided by GRAPPA acceleration techniques. The flip angle was maintained at 80 degrees. Additionally, a high-resolution magnetization-prepared rapid gradient echo sequence (MPRAGE) were used for T1-weighted imaging, featuring voxel dimensions of 1 × 1 × 1 mm³, TR of 2.3 s, TE of 2.98 milliseconds, with a FoV of 192 × 256 × 240 mm³, and sagittal slice orientation with a 9-degree flip angle and an inversion time of 1.1 s.

#### Preprocessing steps

Preprocessing, conducted via SPM12 (Wellcome Trust Center for Neuroimaging, London, UK), comprised realignment, unwarping, co-registration, and normalization. A 4 mm^3^ Gaussian kernel was utilized for smoothing. A composite template was generated from all participants’ high-resolution T1-weighted scans (*n* = 40) for grey-white matter mask (GWM mask) by a probabilistic segmentation approach.

#### Physiological denoising

To include breathing and pulse rate as nuisance regressors in the image analysis using the selective averaging method of Deckers et al. [19], these parameters were recoded via a respiratory belt and pulse oximetry.

#### Laterality

Following our previous study design in PIFP patients we administered the experimental stimulation into the nostril of the affected pain side. Consistent with previous studies [22] and to enable image analysis from only one side, the images of patients with predominantly right-sided pain were mirrored to align with the stimulation site (left) of other patients and HC who were stimulated in the left nostril. Because of usually bilateral brainstem activation after unilateral trigeminal stimulation [20], the stimulation side was considered as a nuisance regressor in further analysis.

### Statistical analysis

General Linear Model (GLM) approach was utilized to derive β-estimates for each participant, which were then utilized in the group-level statistical analysis. These β-estimates represent voxel-wise condition-specific neuronal activity. We employed a hemodynamic response function (HRF) to model the timing and duration of four primary stimuli—ammonia, rose odor, air puff, and visual stimuli—as well as three confounding factors: key presses/assessments, attention tasks, and anticipation phases. These were incorporated as regressors in the GLM by convolving their onset and duration times with the HRF. To account for movement-related variance not corrected during initial realignment steps, we included six additional regressors in the GLM for movement derived from the realignment and unwarp processes. For correcting physiological noise, we added 18 to 20 additional regressors based on each participant’s breathing and pulse data (Expression, Philipps, Best, Netherlands), following the methodology outlined by Deckers et al. [19].

Second-level analysis for the trigeminal painful stimulation across all participants (patients and healthy controls) was performed with one-sample T-test. We applied a threshold for statistical significance uncorrected of *p* < 0.0005 (uncorrected). In more focused analyses, guided by strong prior hypotheses (as noted in our preregistration and previous study [1]), we calculated two-sample t-tests (patients vs. healthy controls), applying an uncorrected statistical threshold of *p* < 0.001 (uncorrected).

The spinal trigeminal nucleus (sTN) was prioritized as the initial processing site for trigeminal pain and has shown to be pivotal involved in the altered trigeminal pain processing in PIFP patients [1], and based on published evidence [1, 13, 20–22] we emphasize this as a significant region of interest (ROI). A small volume correction (6 mm sphere) at previously reported sTN coordinates (x: ±1, y: −46, z: −54) was applied [1], with family-wise error rate (FWE) adjustments confined to this ROI.

To extend our analysis, we incorporated the imaging data from the historical cohort of patients with PIFP from our database [1] and conducted a two-sample T-test to compare them with patients with NFP. This analysis was specifically designed to uncover potential differences in neural activation patterns between these two patient groups, which, despite sharing clinical similarities such as persistent facial pain, may exhibit distinct neurophysiological characteristics. For this analysis, a two-sample t-test was conducted, applying a statistical threshold of *p* < 0.001 (uncorrected).

### Data availability

Researchers meeting the criteria for access to confidential data may access the data upon reasonable request, including the documentation of data access.

## Results

### Patients

Twenty-one patients diagnosed with neuropathic facial pain were recruited for study participation. One participant had to be excluded due to a high amount of movement artefacts in the MRI, resulting in a final sample size of 20 patients for analysis. We also included 20 pain-free control participants, matched by age and sex, using the same experimental protocol. In the patient group 75% of the participants were female, with an average age of 49.9 years (± 12.46 years). On average, patients experienced neuropathic facial pain for 4.7 years, with a range from 4 months to 17 years. All patients exhibited typical symptoms of neuropathic pain, such as sensory loss (negative symptoms) and allodynia or tingling paresthesia (positive symptoms). 16 of the patients were able to identify an iatrogenic triggering event, typically dental procedures such as apicoectomies, tooth extractions, or implant placements. In 4 patients the pain occurred without an identifiable cause such as operation or trauma. Twelve patients took preventatives because of the pain, predominantly amitriptyline or gabapentin and pregabalin. Two patients regularly took non-steroidal anti-inflammatory drugs (NSAIDs). PIFP patients from our earlier study using the identical experimental setup [1]were clinically comparable with the exception of having no somatosensory changes whatsoever. Demographic data can be found in Table 1.

**Table 1:** Demographic data of the study population. Patients fulfilled the ICOP criteria for neuropathic facial pain [3]. In all patients the pain was only on one side and was accompanied by signs of nerve damage in the painful area, such as hypoesthesia, dysesthesia or allodynia

	Neuropathic facial pain patients (*n* = 20)	Healthy controls (*n* = 20)
Female (%)	**15 (75%)**	**15 (75%)**
Mean age (SD) [years]	**49.9 (12.46)**	**50.1 (12.03)**
Pain (data for 20 patients)		
Mean Disease duration (SD) [years]	**4.72 (4.19)**	
Pain intensity (SD) [NRS; 0–10]	**4.9 (1.77)**	
Localized predominantly in the maxilla, n (%)	**6 (30%)**	
Localized predominantly in the mandible, n (%)	**7 (35%)**	
Localized in the maxilla and the mandible, n (%)	**7 (35%)**	

### Behavioral data

The mean intensity ratings (on a 0-100 visual analogue scale) for the nociceptive trigeminal input did not differ significantly (*p* = 0.18) between patients (69.76; CI 61.9,77.62) and HC (64.29; CI 57.44,71.12) as tested with Mann-Whitney.

### Neuroimaging data

#### Main effect trigeminal nociceptive stimulation (patients and healthy controls)

The main effect of trigeminal nociceptive stimulation including all participants revealed activations in various cortical and subcortical regions responsible for central pain and salience processing [1, 11, 20]. Among these structures are the spinal trigeminal nucleus (STN), as well as the thalamus, insula, putamen, and cerebellum. These results reached statistical significance at a threshold of *p* < 0.0005 (uncorrected).

#### NFP patients: alteration in central pain processing (patients vs. healthy control)

Contrasting neuropathic facial pain patients and healthy controls (patients > healthy controls), showed increased activation in subcortical structures such as the insula and the cerebellum to trigeminal nociceptive stimuli in the patient group. Additionally, the sTN (x = 6; y=−45; z=−57) also showed heightened activation compared to the healthy control group at a threshold of *p* < 0.001 (uncorrected) Complete data can be found in Table 2. To test that the sTN region is similar to the increased activation to trigeminal nociception in PIFP patients [1], a small volume correction of the ROI x = 0; y=−46; z=−54 was performed with a sphere of 6 mm showing a significant agreement at the peak level (pFWE-corr_(Peak)_ = 0.037, x = 5; y=−45; z=−57), but not at cluster level (pFWE-corr_(Cluster)_ = 0.634).

**Table 2:** List of brain activations contrasting NFP patients > healthy controls: Two-sample T-test, *n* = 20 per group (condition: pain) threshold *p* < 0.001 (uncorrected, whole volume, GWM masked), extent threshold k > 10 voxels

Name of Region	Cluster Size (voxels)	T-Value (peak)	x	y	z
*Cerebelum_4_5_R*	**17**	**4.09**	**8**	**−47**	**−11**
*Cerebelum_6_L*	**23**	**4.70**	**−30**	**−61**	**−31**
*Cerebelum_6_L*	**11**	**3.77**	**−4**	**−66**	**−23**
*Cerebelum_6_R*	**30**	**4.95**	**9**	**−83**	**−15**
*Cerebelum_6_R*	**46**	**4.36**	**23**	**−74**	**−18**
*Cerebelum_6_R*	**25**	**3.66**	**29**	**−63**	**−21**
*Cerebelum_8_L*	**13**	**3.82**	**−28**	**−42**	**−50**
*Cerebelum_8_R*	**30**	**4.61**	**28**	**−60**	**−45**
*Cerebelum_8_R*	**24**	**4.34**	**36**	**−57**	**−53**
*Cerebelum_9_L*	**36**	**4.17**	**−15**	**−48**	**−53**
*Cerebelum_9_R*	**10**	**4.03**	**15**	**−46**	**−51**
*Cerebelum_Crus1_R*	**33**	**4.24**	**22**	**−80**	**−26**
*Cerebelum_Crus2_L*	**12**	**3.77**	**−42**	**−52**	**−43**
*Cerebelum_Crus2_R*	**21**	**4.19**	**39**	**−63**	**−43**
*Cerebelum_Crus2_R*	**16**	**4.08**	**10**	**−76**	**−36**
*Fusiform_L*	**21**	**5.26**	**−28**	**−54**	**−10**
*Insula_L*	**24**	**4.32**	**−36**	**−1**	**−7**
*Insula_L*	**15**	**3.78**	**−35**	**0**	**−14**
*Insula_L*	**22**	**3.75**	**−27**	**12**	**−13**
*Lingual_R*	**25**	**3.99**	**18**	**−49**	**−4**
*Occipital_Inf_L*	**23**	**4.35**	**−38**	**−77**	**−14**
*Pallidum_L*	**27**	**4.23**	**−19**	**−1**	**−6**
*ParaHippocampal_R*	**18**	**3.95**	**29**	**−3**	**−33**
*Putamen_L*	**14**	**3.93**	**−24**	**12**	**−7**
*Spinal_Trigeminal_N_R*	**27**	**4.92**	**6**	**−45**	**−57**
*Temporal_Mid_L*	**20**	**4.27**	**−55**	**−39**	**−4**
*Temporal_Mid_R*	**11**	**4.22**	**46**	**−2**	**−27**

The inverse contrast (patients < healthy controls) showed no significant results.

#### Contrasting PIFP and NFP patients

**Fig. 1:**
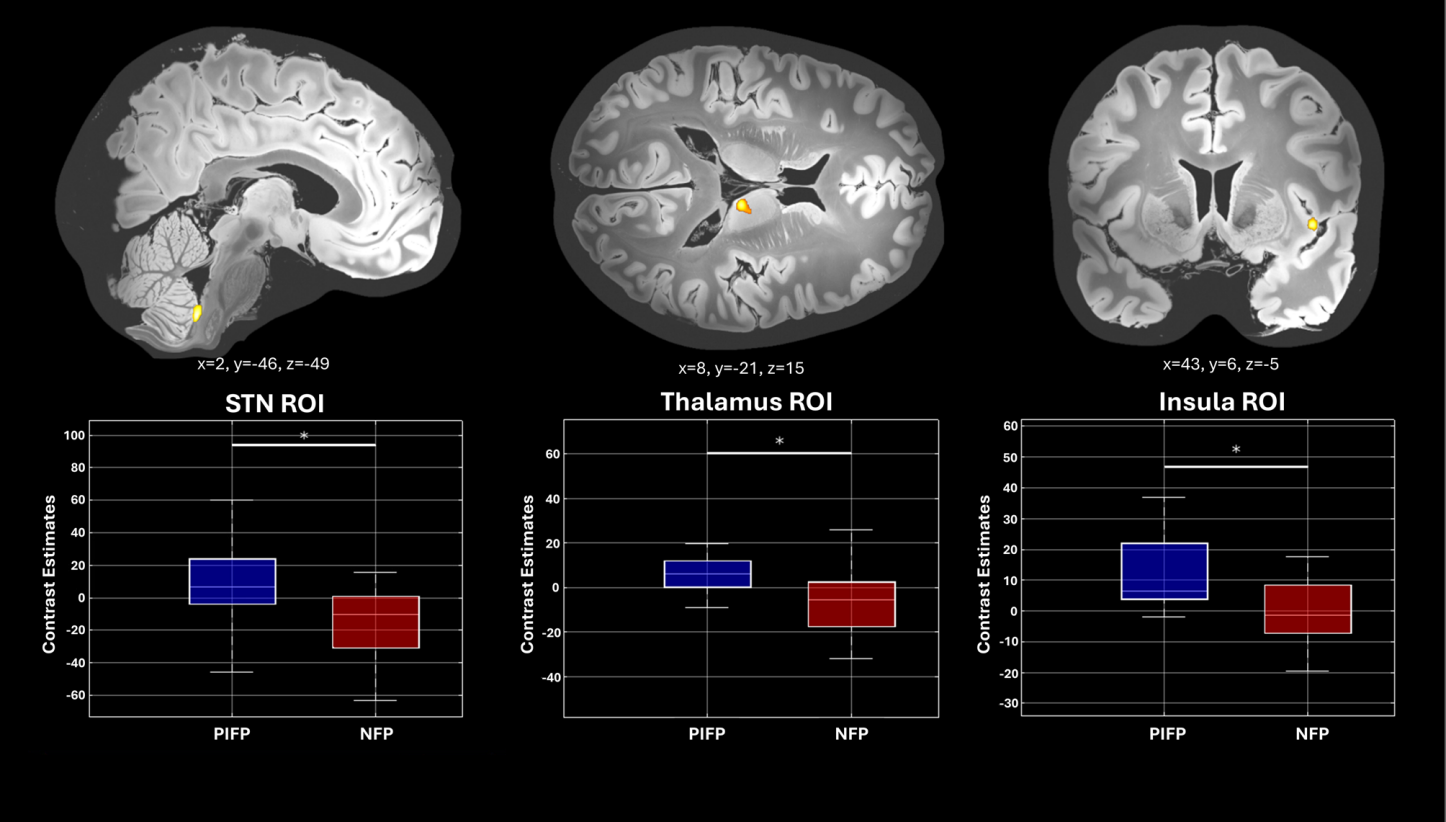
T-score map for the Two-sample T-test second-level contrast between patients with persistent facial pain (PIFP) and patients with neuropathic facial pain (NFP) (PIFP [*n* = 25] > NFP [*n* = 20]). Condition: trigeminal nociceptive pain. Visualization threshold set at *p* < 0.05 (uncorrected, whole Volume GWM masked), with an extent threshold of k = 10 voxels. Upper row: Higher activation (PIFP > NFP) is shown for three different regions: STN, Thalamus, Insula. The coordinates are displayed, with the exact layer indicated in bold. Lower row: Parameter estimates of the neuronal activity for the three different regions (defined as ROI) for PIFP and NFP are shown as boxplots. The central line represents the median in boxplots, the box edges indicate the 25th and 75th percentiles (interquartile range), and the whiskers extend to show the range of the data within 1.5 times the interquartile range. Outliers are not shown. Significance is marked with an asterisk and a line, tested by a two-sample test (2-sided, *p* < 0.001)

**Fig. 2:**
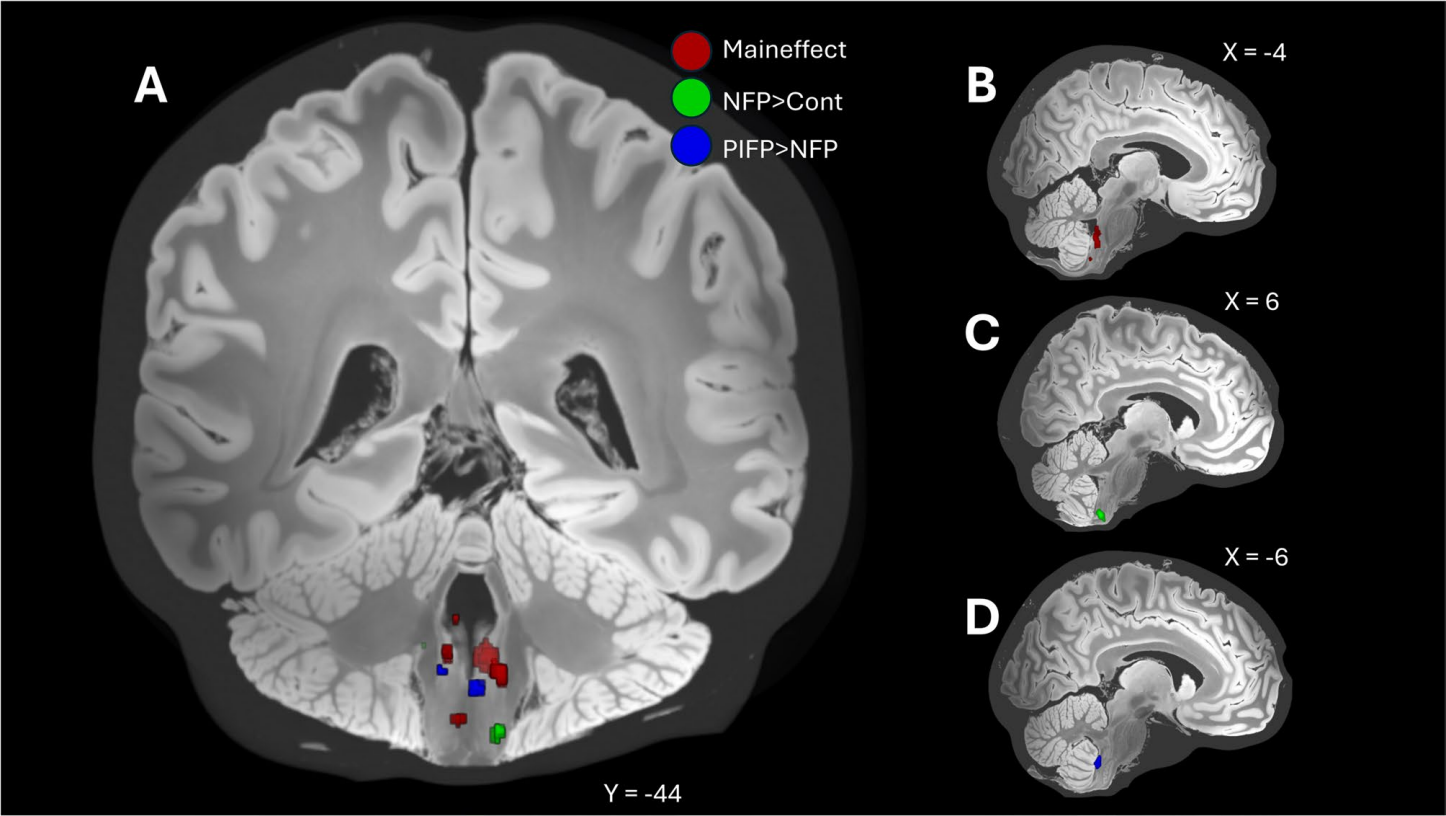
**A** T-score map illustrating different activations among the coronal brainstem: Red: Main-effect across all study participants (NFP patients and healthy controls, *n* = 40). Condition: trigeminal nociceptive pain. Visualization threshold set at *p* < 0.001 (uncorrected, brainstem masked), Green: Patients with neuropathic facial pain (NFP) > healthy controls. Condition: trigeminal nociceptive pain. Visualization threshold set at *p* < 0.001 (uncorrected, brainstem masked) Blue: PIFP patient group compared to the NFP patients (PIFP _[*n*=25]_ > NFP_[*n*=20]_) Condition: trigeminal nociceptive pain. Visualization threshold set at *p* < 0.001 (uncorrected, brainstem masked). For further details, refer to Table 3. Right Side: Above mentioned contrasts shown individually to visualize different heights on the sagittal slice. **B** Main-effect across all study participants (NFP patients and healthy controls, *n* = 40). Condition: trigeminal nociceptive pain. Visualization threshold set at *p* < 0.01 (uncorrected, brainstem masked), **C** Patients with neuropathic facial pain (NFP) > healthy controls. Condition: trigeminal nociceptive pain. Visualization threshold set at *p* < 0.01 (uncorrected, brainstem masked) **D** PIFP patient group compared to the NFP patients (PIFP _[*n*=25]_ > NFP_[*n*=20]_) Condition: trigeminal nociceptive pain. Visualization threshold set at *p* < 0.01

Contrasting PIFP with NFP patients (PIFP > NFP), showed increased activation in subcortical regions involved in pain processing like thalamus, insula, temporal gyrus and the cerebellum at a threshold of *p* < 0.001 (uncorrected) (see Table 3; Figs. 1 and 2). Using an additional interaction analysis (PIFP[patients]-PIFP[control]) > (NFP[patients]-NFP[control]) showed the same result. The reverse contrast (PIFP < NFP) showed no significant results.

**Table 3:** List of brain activations contrasting PIFP patients_[*n*=25]_ > NFP patients_[*n*=20]_ : Two-sample T-test (condition: pain) threshold *p* < 0.001 (uncorrected, whole volume), extent threshold k > 10 voxels

Name of Region	Cluster Size (voxels)	T-Value (peak)	x	y	z
Left Brainstem	***46***	***5.20***	***−11***	***−28***	***−23***
Thalamus_R	***67***	***4.79***	***16***	***−10***	***0***
Thalamus_R	***54***	***4.77***	***8***	***−21***	***15***
Cerebellum_4_5_L	***41***	***4.69***	***−10***	***−47***	***−9***
Right Cerebrum	***12***	***4.67***	***30***	***−51***	***27***
Cerebellum_4_5_L	***69***	***4.54***	***−12***	***−60***	***−16***
Right Brainstem	***18***	***4.45***	***11***	***−28***	***−16***
Temporal_Sup_L	***47***	***4.35***	***−61***	***−1***	***−6***
Left Cerebrum	***40***	***4.32***	***−26***	***−43***	***26***
Cerebellum_4_5_L	***35***	***4.25***	***−4***	***−62***	***−15***
Cuneus_R	***58***	***4.18***	***26***	***−61***	***19***
Cerebellum_Crus1_L	***23***	***4.09***	***−51***	***−50***	***−35***
Right Cerebrum	***24***	***4.08***	***21***	***−28***	***19***
Temporal_Mid_L	***23***	***4.06***	***−55***	***−25***	***−4***
ParaHippocamapal_R	***95***	***4.05***	***22***	***−17***	***−24***
Vermis_8	***30***	***4.04***	***5***	***−63***	***−36***
Thalamus_L	***25***	***3.98***	***−11***	***−23***	***18***
Putamen_R	***16***	***3.88***	***31***	***2***	***7***
Insula_R	***10***	***3.88***	***43***	***6***	***−5***
Cerebellum_6_R	***44***	***3.83***	***8***	***−64***	***−20***
Temporal_Sup_L	***23***	***3.79***	***−40***	***−4***	***−14***
Temporal_Pole_Sup_R	***10***	***3.67***	***53***	***10***	***−15***
ParaHippocampal_R	***10***	***3.56***	***27***	***−28***	***−27***
Spinal_Trigeminal_N_R	***14***	***3.55***	***2***	***−46***	***−49***

## Discussion

Our study revealed two main findings: First, in response to experimental painful trigeminal stimulation, NFP patients exhibit stronger activation than healthy volunteers in pain-processing areas, including the bilateral insular cortex, para-hippocampal region, basal ganglia, cerebellum, bilateral mid-temporal lobes, and spinal trigeminal nucleus. This is in line with an earlier study on diabetic neuropathic pain [23].

The second finding is that patients with PIFP activate the same central pain transmitting structures even more and to the extent that PIFP and NFP are clearly distinguishable when using functional neuroimaging.

The pain levels due to our experiment where equal in NFP patients and healthy controls and indeed similar to our previous migraine study [24] and also to our previous study in PIFP patients [1] which all used the same experimental design. This implies that the perceived level of experimental pain does not explain the difference in pain related activation in NFP patients. It is likely that the preexistent facial pain of our patients, which was also present on the day of the examination, is caused by or at least contributes to the altered pain transmission of NFP patients when compared to pain-free healthy controls. A similar overactivity of the brain areas including the spinal trigeminal nucleus has recently been observed by our group in PIFP patients and has been attributed to central sensitization as either a cause or consequence of persistent facial pain [1].

Although the net-effect - altered central nociceptive activation in specific brain areas- is similar in both pain syndromes, the underlying cause appears fundamentally different. All NFP patients in our study suffered from peripheral nerve damage as the cause of their neuropathic pain. According to definitions, this peripheral damage accounts for the pain experienced [25]. The constant disease-related pain in NFP patients merges with the short-lasting experimental nociceptive input in our study, both activating the trigeminal nucleus, the first central relay station for trigeminal pain. Given that central sensitization is defined as “an amplification of neural signaling within the central nervous system that elicits pain hypersensitivity” [26] our data may suggest that NFP patients exhibit a central sensitization as the major underlying mechanism of the constant pain.

PIFP is frequently linked to minor surgical procedures or dental or otolaryngologic interventions [5]. These procedures are often either described as the triggering event or as an attempt to treat the pain [27–29]. Consequently, in some cases, it is impossible to distinguish whether the clinical neuropathic signs are the cause or the consequence of the treatment. This overlap raises debates on whether PIFP and NFP are independent conditions or lie at opposite ends of a continuum [5]. To distinguish the true idiopathic facial pain syndromes such as PIFP from neuropathic pain, it will be essential to adhere to internationally accepted classification of facial pain [3] and ideally include intraoral quantitative sensory testing (QST) to detect subclinical nerve damage [30, 31].

In our study contrasting PIFP and NFP patients, we adhered strictly to international classification standards: By definition, PIFP patients showed no clinical signs of nerve damage whatsoever and exhibited strictly unilateral constant pain. All our NFP patients experienced similar persistent pain with a burning character and additionally in the same unilateral facial area exhibited clinical signs of nerve damage, such as hypoesthesia, dysesthesia, or allodynia. Most NFP patients had a history of peripheral operations or trauma associated with nerve damage and pain.

Contrasting the two patient groups – PIFP vs. NFP- revealed that despite similar “overactive” trigeminal nucleus profiles, PIFP and NFP patients could additionally be distinguished by different activation patterns of subcortical brain areas, including the brainstem, the contralateral thalamus, bilateral insular cortex, and bilateral facial area of the somatosensory cortex. This finding suggests that PIFP and NFP are distinct entities, with PIFP not readily explained by “occult” neuronal damage, implying they are not merely “ends of the same continuum” as currently thought. This raises the question of whether persistent pain in PIFP patients might be caused by an overreactive (sensitized) brain rather than peripheral nociceptive input, representing a nociplastic pathophysiology. We suggest that such an overactivity of pain transmitting areas without any clinical signs of neuropathic origin is indeed a visualization of a nociplastic pain syndrome [32]. We acknowledge that our data challenge the current IASP definitions, that to qualify for a nociplastic pain syndrome a gain in function (i.e., clinical signs of pain hypersensitivity) is needed [33]. In a very thorough study using quantitative sensory testing, blink reflexes and somatotopy of the primary somatosensory cortex (SI) to tactile input from the pain area [34], no significant differences were found between PIFP patients and healthy controls. Consequently it was suggested that “PIFP is maintained by mechanisms which do not involve somatosensory processing of stimuli from the pain area” [34]. Our data suggest a central mismatch in pain transmitting structures which maintain the persistent pain without peripheral input (nociceptive) or damage (neuropathic).

Our field of view allows for investigating brain pain transmission circuitry and in the same frame the trigeminal nucleus as the first central relay station of incoming trigeminal nociceptive signals. Chronic facial pain syndromes may thus be ideal candidates for investigating pain chronicity and neuroplastic pain. Our data indicate that chronic facial pain results in overactivated transmission of pain signals on brainstem level where trigeminal first-order neurons synapse with second-order neurons. Whether this overactivation is specific to facial pain syndromes or merely visualizes chronic pain in terms of neuroplastic changes remains unresolved. Future studies should employ local anesthesia or anesthetic nerve blocks to differentiate patients whose pain diminishes with local anesthesia from those whose pain persists even when the dermatome is anesthetic [35]. In the latter case, a truly central origin of facial pain is likely, and investigating these patients with brainstem imaging will be crucial to understanding central pain syndromes arising from peripheral origin.

### Limitations

We note, that stimulating the nerve endings in the nasal cavity excites the 1 st and 2nd branch of the trigeminal nerve. Any changes in contrasting both groups shows therefore that the trigeminal system rather than a damaged trigeminal nerve fibre is the source of the pain. As noted above, The IASP definition of neuropathic pain distinguishes “probable” from “definitive” neuropathy. We note that including “probable” NFP is not a limitation though. Probable and definitive neuropathic pain are clinically identical and are both counted as neuropathic pain. The difference being that to use the word “definitive” requires ancillary tests as an external, non-clinical reference. A more important issue is the relatively small number of patients investigated. The complexity of neuroimaging studies does not allow large number of patients to be assessed. Still, it needs pointing out that the number of assessed patients is a key factor in determining reproducibility and the strength of the conclusion drawn. We certainly agree that our findings need to be replicated. That said, we found a difference between both cohorts, and this suggests that both cohorts may indeed have different pathophysiological underpinnings. One could argue that we should have reported corrected p-values. In our MRI experiment, voxels are 1.25 × 1.25 × 2.5 mm³. The spinal trigeminal nucleus (sTN) has just 8–12 voxels, corresponding to a physical volume of approximately 30–40 mm³. Cluster-level FWE corrections at pFWE < 0.05 would typically demand clusters of greater than 100 mm³ to be significant under Gaussian random‐field theory. Given the spatial resolution of our data and the limited voxel count comprising the sTN and other brainstem nuclei, conventional whole‐brain, cluster‐level FWE corrections would not only be arbitrary but also counterproductive [1, 11, 22]. Regarding our above mentioned suggestion of a follow-up study (local anaesthesia or aesthetic nerve blocks), such a study would focus on PIFP patients alone and would be an ideal opportunity to differentiate PIFP patients with an assumable peripheral pain origin from patients with a true central pain origin. We did not have an ethics vote for such a study at the time when we preregistered this experiment in 2020. The present study now allows a clear hypothesis and thus a hypothesis-driven follow-up study.

## Supplementary Information

Supplementary Material 1.

Supplementary Material 2.

Supplementary Material 3.

## Data Availability

Researchers meeting the criteria for access to confidential data may access the data upon reasonable request, including the documentation of data access.
