# Supplementary material for: Visualizing nociplastic pain: functional hyperexcitability in neuropathic and idiopathic facial pain syndromes
Source: J Headache Pain. 2025 Oct 13;26(1):211. doi: 10.1186/s10194-025-02133-w (PMC12516831; doi:10.1186/s10194-025-02133-w)
Supplement: Supplementary file 1 — Supplementary Material 1. [file 10194_2025_2133_MOESM1_ESM.docx]

|  | Neuropathic facial pain patients (n=20) | | *Persistent idiopathic facial pain* (n=25) | | Healthy controls (n=20) |
| --- | --- | --- | --- | --- | --- |
| Female (%) | 15 (75%) | *23 (92%)* | | 15 (75%) | |
| Age – Mean (SD) [years] | 49.9 (12.46) | *49.2 (13.0)* | | 50.1 (12.03) | |
| Pain history |  | *n=1 missing* | |  | |
| Disease duration (SD) [years] | 4.72 (4.19) | *7.9 (7.4)* | |  | |
| Pain intensity (SD) [NRS; 0-10] | 4.9 (1.77) | *3.8 (2.2)* | |  | |
| Localized predominantly  in the maxilla, n (%) | 6 (30%) | *13 (52%)* | |  | |
| Localized predominantly  in the mandible, n (%) | 7 (35%) | *3 (12%)* | |  | |
| Localized in the maxilla  and the mandible, n (%) | 7 (35%) | *8 (32%)* | |  | |

**Table 1:** Demographic data of this study population and previous study on persistent idiopathic facial pain patients (shown in italics, (Ziegeler et al., 2021)). Patients fulfilled the ICOP criteria for neuropathic pain and persistent idiopathic facial pain (“International Classification of Orofacial Pain, 1st edition (ICOP),” 2020). In all NFP patients the pain was only on one side and was accompanied with a sign of nerve damage in the painful area, such as hypoesthesia, dysesthesia or allodynia.
