# Supplementary material for: Visualizing nociplastic pain: functional hyperexcitability in neuropathic and idiopathic facial pain syndromes
Source: J Headache Pain. 2025 Oct 13;26(1):211. doi: 10.1186/s10194-025-02133-w (PMC12516831; doi:10.1186/s10194-025-02133-w)
Supplement: Supplementary file 2 — Supplementary Material 2. [file 10194_2025_2133_MOESM2_ESM.docx]

**Table 3**

| **Name of Region** | **Cluster Size (voxels)** | | **T-Value (peak)** | **x** | **y** | **z** |
| --- | --- | --- | --- | --- | --- | --- |
| Cerebelum_4_5_R | | 17 | 4.09 | 8 | -47 | -11 |
| Cerebelum_6_L | | 23 | 4.70 | -30 | -61 | -31 |
| Cerebelum_6_L | | 11 | 3.77 | -4 | -66 | -23 |
| Cerebelum_6_R | | 30 | 4.95 | 9 | -83 | -15 |
| Cerebelum_6_R | | 46 | 4.36 | 23 | -74 | -18 |
| Cerebelum_6_R | | 25 | 3.66 | 29 | -63 | -21 |
| Cerebelum_8_L | | 13 | 3.82 | -28 | -42 | -50 |
| Cerebelum_8_R | | 30 | 4.61 | 28 | -60 | -45 |
| Cerebelum_8_R | | 24 | 4.34 | 36 | -57 | -53 |
| Cerebelum_9_L | | 36 | 4.17 | -15 | -48 | -53 |
| Cerebelum_9_R | | 10 | 4.03 | 15 | -46 | -51 |
| Cerebelum_Crus1_R | | 33 | 4.24 | 22 | -80 | -26 |
| Cerebelum_Crus2_L | | 12 | 3.77 | -42 | -52 | -43 |
| Cerebelum_Crus2_R | | 21 | 4.19 | 39 | -63 | -43 |
| Cerebelum_Crus2_R | | 16 | 4.08 | 10 | -76 | -36 |
| Fusiform_L | | 21 | 5.26 | -28 | -54 | -10 |
| Insula_L | | 24 | 4.32 | -36 | -1 | -7 |
| Insula_L | | 15 | 3.78 | -35 | 0 | -14 |
| Insula_L | | 22 | 3.75 | -27 | 12 | -13 |
| Lingual_R | | 25 | 3.99 | 18 | -49 | -4 |
| Occipital_Inf_L | | 23 | 4.35 | -38 | -77 | -14 |
| Pallidum_L | | 27 | 4.23 | -19 | -1 | -6 |
| ParaHippocampal_R | | 18 | 3.95 | 29 | -3 | -33 |
| Putamen_L | | 14 | 3.93 | -24 | 12 | -7 |
| Spinal_Trigeminal_N_R | | 27 | 4.92 | 6 | -45 | -57 |
| Temporal_Mid_L | | 20 | 4.27 | -55 | -39 | -4 |
| Temporal_Mid_R | | 11 | 4.22 | 46 | -2 | -27 |
