# Supplementary material for: Visualizing nociplastic pain: functional hyperexcitability in neuropathic and idiopathic facial pain syndromes
Source: J Headache Pain. 2025 Oct 13;26(1):211. doi: 10.1186/s10194-025-02133-w (PMC12516831; doi:10.1186/s10194-025-02133-w)
Supplement: Supplementary file 3 — Supplementary Material 3. [file 10194_2025_2133_MOESM3_ESM.docx]

**Table 3**

| **Name of Region** | **Cluster Size**  **(voxels)** | **T-Value**  **(peak)** | **x** | **y** | **z** |
| --- | --- | --- | --- | --- | --- |
| Left Brainstem | *46* | *5.20* | *-11* | *-28* | *-23* |
| Thalamus_R | *67* | *4.79* | *16* | *-10* | *0* |
| Thalamus_R | *54* | *4.77* | *8* | *-21* | *15* |
| Cerebellum_4_5_L | *41* | *4.69* | *-10* | *-47* | *-9* |
| Right Cerebrum | *12* | *4.67* | *30* | *-51* | *27* |
| Cerebellum_4_5_L | *69* | *4.54* | *-12* | *-60* | *-16* |
| Right Brainstem | *18* | *4.45* | *11* | *-28* | *-16* |
| Temporal_Sup_L | *47* | *4.35* | *-61* | *-1* | *-6* |
| Left Cerebrum | *40* | *4.32* | *-26* | *-43* | *26* |
| Cerebellum_4_5_L | *35* | *4.25* | *-4* | *-62* | *-15* |
| Cuneus_R | *58* | *4.18* | *26* | *-61* | *19* |
| Cerebellum_Crus1_L | *23* | *4.09* | *-51* | *-50* | *-35* |
| Right Cerebrum | *24* | *4.08* | *21* | *-28* | *19* |
| Temporal_Mid_L | *23* | *4.06* | *-55* | *-25* | *-4* |
| ParaHippocamapal_R | *95* | *4.05* | *22* | *-17* | *-24* |
| Vermis_8 | *30* | *4.04* | *5* | *-63* | *-36* |
| Thalamus_L | *25* | *3.98* | *-11* | *-23* | *18* |
| Putamen_R | *16* | *3.88* | *31* | *2* | *7* |
| Insula_R | *10* | *3.88* | *43* | *6* | *-5* |
| Cerebellum_6_R | *44* | *3.83* | *8* | *-64* | *-20* |
| Temporal_Sup_L | *23* | *3.79* | *-40* | *-4* | *-14* |
| Temporal_Pole_Sup_R | *10* | *3.67* | *53* | *10* | *-15* |
| ParaHippocampal_R | *10* | *3.56* | *27* | *-28* | *-27* |
| Spinal_Trigeminal_N_R | *14* | *3.55* | *2* | *-46* | *-49* |
